# Anxiety, depression, resilience and self-esteem in individuals with cardiovascular diseases**^1^**

**DOI:** 10.1590/1518-8345.1405.2836

**Published:** 2016-11-28

**Authors:** Isabela Gonzales Carvalho, Eduarda dos Santos Bertolli, Luciana Paiva, Lidia Aparecida Rossi, Rosana Aparecida Spadoti Dantas, Daniele Alcalá Pompeo

**Affiliations:** 2 RN.; 3 PhD, RN, Hospital das Clínicas, Universidade Federal do Triângulo Mineiro, Uberaba, MG, Brazil.; 4 PhD, Full Professor, Escola de Enfermagem de Ribeirão Preto, Universidade de São Paulo, PAHO/WHO Collaborating Centre for Nursing Research Development, Ribeirão Preto, SP, Brazil.; 5 PhD, Associate Professor, Escola de Enfermagem de Ribeirão Preto, Universidade de São Paulo, PAHO/WHO Collaborating Centre for Nursing Research Development, Ribeirão Preto, SP, Brazil.; 6 PhD, Adjunct Professor, Departamento de Enfermagem Especializada, Faculdade de Medicina de São José do Rio Preto, São José do Rio Preto, SP, Brazil.

**Keywords:** Adaptation, Self Concept, Cardiovascular Diseases, Anxiety, Depression, Nursing

## Abstract

**Objectives:**

to analyze the relationship between anxiety and depression symptoms, resilience and self-esteem with sociodemographic and clinical characteristics; correlate resilience and self-esteem with age and duration of the disease; check associations between anxiety and depression with measures of resilience and self-esteem among individuals with cardiovascular diseases.

**Method:**

correlational study conducted in a large university hospital in the interior of the state of São Paulo, Brazil. The population was composed of adult inpatients with cardiovascular diseases. A non-probabilistic consecutive sample was composed of 120 patients. Variables of interest were assessed using the Hospital Anxiety and Depression Scale, Resilience Scale, and Rosenberg Self-Esteem Scale.

**Results:**

anxiety and depression symptoms were present in 32.5% and 17.5% of the patients, respectively, and were associated with the female sex (p = 0.002; p = 0.022). Manifestations of depression were associated with the presence of comorbidities (p = 0.020). More resilient patients did not present depression symptoms (p < 0.001) and anxious women were more resilient (p = 0.042). The highest scores regarding self-esteem were present in patients with anxiety and depression. Men presented higher resilience and lower self-esteem compared to women.

**Conclusion:**

patients with anxiety and depression were less resilient but presented higher self-esteem.

## Introduction

Noncommunicable diseases are the primary cause of death in the world, leading to a high rate of early deaths, loss of quality of life, and limiting work and leisure activities, in addition to economically impacting families, communities and society in general, aggravating inequality and poverty^1^. 

Similar to the contexts of other countries, in Brazil noncommunicable diseases represent a major health problem accounting for 72% of deaths, especially those caused by chronic cardiovascular, circulatory and respiratory conditions, affecting individuals of all socioeconomic strata, though more intensely impacting those belonging to vulnerable groups, such as the elderly and those with low income and education levels^1^.

Individuals with cardiovascular diseases are exposed to physical, psychological and social discomfort related to their treatment, which influences their ability to adapt to a new lifestyle^2^. Additionally, this burden may cause changes in family dynamics given the patient's greater care demands.

Self-care management in the case of chronic conditions is desired to prevent disease-related complications. One study conducted with 628 patients with symptomatic heart failure verified associations between high levels of self-care and improved quality of life and lower hospitalization rates. Additionally, the more comorbidities presented, the lower the involvement of patients with the management of their treatment^2^.

The ability of patients to assume responsibility for their own treatment is affected by interrelated personal characteristics such as motivation, self-efficacy and resilience. The identification of efficient ways to promote the development of these characteristics in this population can improve patients' coping strategies and prepare them to properly dealing with a chronic disease^3^.

Resilience is defined as a process of negotiation, management, and adaptation to significant sources of stress or trauma^4^; it is an individual's ability to adjust to adversities, keep balance and carry on with life in a positive way^5^. 

Resilient people are less susceptible to diseases^6^ and have a greater ability to relieve pressure caused by a disease's negative impact^7^. One study conducted in Sweden reports that low levels of resilience during adolescence were associated with increased risk of heart disease during adult life^6^.

Less resilient individuals are possibly more susceptible to stress and present poor coping strategies when facing adversity, which may generate anxiety, depression, anger, impulsiveness, and low self-esteem.

Therefore, nursing workers should reflect upon and develop proposals for acquiring a more detailed anamnesis involving not only the physical aspects of the disease, but also the psychosocial ones. Little attention, however, has been paid to the identification of these characteristics in hospital settings. 

A literature review identified studies assessing resilience in patients with congenital cardiovascular diseases^8^ ^-^ ^9^ and congestive heart failure^10^. There are few studies addressing resilience in this population using scales validated in Brazil, especially resilience related to self-esteem, anxiety or depression. These results can support the strengthening of multidisciplinary strategies focusing on increased resilience and emotional skills to help patients cope with stress arising from heart disease.

Therefore, this study's objectives were: 1) analyze potential associations between anxiety and depression with sociodemographic and clinical characteristics; 2) verify association between measures of resilience and self-esteem and sociodemographic and clinical variables; 3) correlate measures of resilience and self-esteem with age and duration of disease; 4) analyze associations between anxiety and depression symptoms with measures of resilience and self-esteem among individuals with cardiovascular diseases.

## Method

This correlational, cross-sectional study was conducted in a 77-bed Clinical and Surgical Hospitalization Unit with a cardiovascular specialty of a university hospital in the interior of the state of São Paulo, Brazil. This hospital is a large facility linked to 102 cities in the northwest region of the state of São Paulo, with a total of 629 beds, having 53,000 appointments per month, and performing 262 transplantations in 2015, including heart transplantations.

The population was composed of patients with cardiovascular diseases of a clinical or surgical etiology, admitted into the aforementioned hospital, regardless of sex, aged 18 years old or older. Exclusion criteria were: not being able to communicate verbally or not having the cognitive condition necessary to participate in the study, which was verified by the patient's ability to report his/her own age or date of birth, address, and the weekday and day of month. The participants who met the inclusion criteria were selected according to non-probabilistic consecutive sampling (n = 120) and data were collected from October 1^st^ 2014 to January 31^st^ 2015.

Four instruments were used to collect data, namely: sociodemographic characterization (sex, age, having a spouse/partner, education, income and occupation); the Hospital Anxiety and Depression Scale (HADS)^11^; Resilience Scale^5^ ^,^ ^12^; and the Rosenberg Self-esteem Scale (RSES)^13^.

HADS is easy to use and can be quickly applied. It can be either self-reported or completed by an interviewer. HADS has 14 items; seven items assess anxiety (HADS-A) and seven assess depression (HADS-D). Each item has four possible answers on a scale from 0 to 3, with a total score of 21 points for each subscale; the higher the score, the higher the presence of anxiety (HADS-A) or depression (HADS-D) symptoms^11^. 

The Resilience Scale measures levels of psychosocial adaptation when facing important life events. It has 25 items positively described with a Likert scale ranging from 1 (totally disagree) to 7 (totally agree). It is subdivided into three dimensions: 1) Hardiness of actions and values: 14 items involve actions related to energy, persistence, discipline, value conceptions, actions directed to giving meaning to life such as friendship, personal accomplishment, satisfaction and meaning of life; 2) Independence and determination: six items address the ability to solve difficult situations, to deal with various situations at the same time, accept adversity and situations that cannot be changed; 3) Self-confidence: five items focus on the belief that a person can handle her/his problems and that doing so depends more on her/him than on others, doing things against one's own will but keeping interest focused on things that matter^12^. This scale's scores range from 25 to 175 points; high scores indicate high resilience^12^. 

RSES^13^ is composed of 10 items with four alternates for answers: 1 strongly agree, 2 agree, 3 disagree, 4 strongly disagree. A measure of self-esteem is obtained by summing up the scores obtained in the scale's items, then recoding five items with reverse scores. The sum of these scores may range from 10 to 40; higher scores indicate higher self-esteem^13^. 

All the scales were translated and adapted to Brazil and present satisfactory internal consistency measured through Cronbach's alpha: HADS-A: 0.68 and HADS-D: 0.77^11^; Resilience Scale: 0.80^12^; RSES: 0.98^13^.

Data collection was initiated a census of patients of the hospitalization ward to confirm heart disease. After receiving clarification regarding the study and consenting to take part in the study, participants signed free and informed consent forms and the interview was then verbally applied by an interviewer. 

Data were processed and analyzed using Minitab 17 (Minitab Inc) and Statistica 10 (SatatSoft Inc). Measures of position (mean and median) and variability (standard deviation) were used. 

Qualitative data were associated by applying the Chi-square test. The age of patients and duration of the disease were compared with sampling characterization variables, clinical variables, and risk factors by applying the t-test for independent samples (when two sampling groups are compared) and Variance Analysis (ANOVA) (when more than two sampling groups are compared).

Person's correlation test was used to analyze continuous variables normally distributed (resilience, self-esteem, age, and duration of disease). The significance level adopted was 0.05.

The study was conducted according to Brazilian and international ethical standards regulating research involving human subjects and was approved under protocol No. 697.946.

## Results

Of the 120 individuals with cardiovascular diseases, 67 (55.8%) were male and 53 (44.2%) were female; age ranged from 26 and 88 years old, with a mean of 58.3 years and a standard deviation of 12.2 years. Most were married (n = 71; 59.2%); 49 individuals (40.8%) had an occupation, while most did not work because they were either on leave, retired or did not have a job (n = 71; 59.2%).

Coronary heart disease was the medical diagnosis of 58 patients (48.3%), followed by congestive heart failure (n = 43; 35.8%) and valvular heart disease (n = 19;15.8%). Almost half of the individuals reported having the disease for more than five years (48.3%); most reported no comorbidities (n = 73; 60.8%) and that the disease interferes in their daily living activities (n = 85; 70.8%).

In regard to lifestyle, most patients reported no smoking (n = 103; 85.5%), no consumption of alcohol (n = 97; 80.8%) and having a personality prone to stress (n = 73; 60.8%).

Anxiety and depression symptoms were presented by 32.5% and 17.5% of the patients, respectively. When these symptoms were analyzed according to sex, women presented more anxiety and depression symptoms than men; these associations were statistically significant (p =0.002 and p = 0.022, respectively). No associations were found between anxiety and depression symptoms and having a partner/spouse or level of education (Table 1).

**Table 1:** Anxiety and depression symptoms presented by the participants according to sex, education, and whether the individual had a partner/spouse. São José do Rio Preto, São Paulo, Brazil, 2014-2015

Variables	Anxiety			Depression	
	No	Yes		No	Yes
Sex	81 (67.50%)	39 (32.50%)		99 (82.50%)	21 (17.50%)
Female	28 (34.57%)	25 (64.10%)		39 (39.39%)	14 (66.67%)
Male	53 (65.43%)	14 (35.90%)		60 (60.61%)	7 (33.33%)
P value*	0.002			0.022	
Education	78 (70.27%)	33 (29.73%)		91 (81.98%)	20 (18.02%)
No education	10 (62.50%)	6 (37.50%)		12 (75.00%)	4 (25.00%)
Primary and middle school	54 (70.13%)	23 (29.87%)		63 (81.82%)	14 (18.18%)
High school	8 (66.67%)	4 (33.33%)		10 (83.33%)	2 (16.67%)
College	6 (100%)	0 (0.00%)		6 (100%)	0 (0.00%)
P value*	0.191			0.410	
Has a partner/spouse	81 (67.50%)	39 (32.50%)		99 (82.50%)	21 (17.50%)
Yes	46 (64.79%)	25 (35.21%)		60 (84.51%)	11 (15.49%)
No	35 (71.40%)	14 (28.60%)		39 (79.60%)	10 (20.40%)
P value*	0.445			0.486	

* P value concerning Chi-square test

The means and standard deviations of the measures for resilience and self-esteem according to sex, education, and whether the individual had a partner/spouse are presented in Table 2. Statistically significant differences were found only between the means of the scales for resilience (p = 0.027) and self-esteem (p = 0.031) in regard to the sex of the participants. Men presented higher scores on the resilience scale, while women presented higher self-esteem scores.

**Table 2:** Measures of resilience and self-esteem according to sex, education and the presence of partner/spouse. São José do Rio Preto, São Paulo, Brazil, 2014-2015

Variables	Resilience		Self-esteem
	Mean [SD (Median)]		Mean [SD (Median)]
Sex			
Female (n = 53)	127.79[11.26 (130.00)]		20.79[2.08 (21.00)]
Male (n = 67)	132.30[10.52 (135.00)]		19.92[2.24 (20.00)]
P value*	0.027		0.031
Education			
No education (n = 16)	125.56[11.18 (126.50)]		20.87[1.25 (21.00)]
Primary and middle school (n = 77)	129.75[11.08 (130.00)]		20.31[2.39 (20.00)]
High school (n = 12)	133.75[12.12 (135.00)]		20.08[2.23 (20.00)]
College (n = 6)	135.17[10.32 (138.50)]		19.50[2.07 (19.50)]
P value^†^	0.166		0.591
Has a partner/spouse			
Yes (n = 71)	130.92[11.22 (132.00)]		20.48[2.17 (20.00)]
No (n = 49)	129.43[10.81 (129.00)]		20.06[2.25 (20.00)]
P value*	0.468		0.315

p* concerning t-test for independent samples; p^†^ concerning Analysis of Variance (ANOVA)

Education and the presence of a partner/spouse did not significantly influence resilience or self-esteem.

Table 3 presents the results from the test of association between anxiety and depression symptoms with the following variables: presence of comorbidities (in addition to the cardiovascular disease); smoking and alcohol consumption; regardless of sex. The only statistically significant association was between depression symptoms and comorbidities (p = 0.020).

**Table 3:**
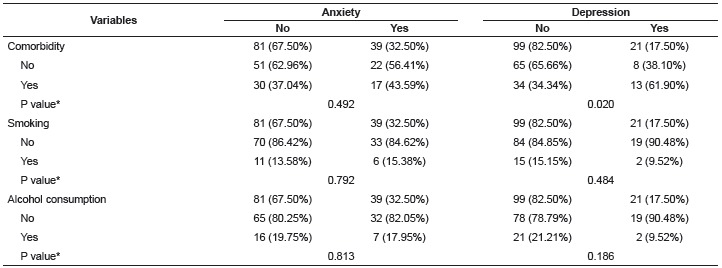
Anxiety and depression symptoms among the participants, according to other comorbidities, smoking and alcohol consumption. São José do Rio Preto, São Paulo, Brazil, 2014-2015

* P value concerning Chi-square test

Patient's age and duration of the disease did not influence anxiety (p = 0.179; p = 0.833) or depression symptoms (p = 0.861; p = 0.855) among the patients under study (P value concerning t-test for independent tests samples at p < 0.05).

There was no association of clinical variables (medical diagnoses and comorbidities) and risk factors (smoking and alcohol consumption) with the resilience and self-esteem scores.

Age and duration of disease were not correlated with resilience and self-esteem scores. The values concerning correlation between resilience and age (r= -0.129, p = 0.161), resilience and duration of the disease (r = 0.069, p = 0.454), self-esteem and age (r = 0.091, p = 0.324), self-esteem and duration of the disease (r = 0.136, p = 0.138), suggest a lack of significant correlation between the analyzed variables (p > 0.050).

Tables 4 and ^5^show that women with symptoms of anxiety (p = 0.042) were significantly less resilient, while anxiety was not a preponderant factor (p = 0.377) among men to the extent of causing significant differences in resilience, though participants of both sexes with depression symptoms were less resilient (p<0.001; p<0.001). No relationship was found between self-esteem and depression for either sex (p = 0.050; p = 0.117). 

**Table 4:** Mean±standard deviation (median) of the female participants' resilience and self-esteem scores in regard to anxiety and depression symptoms. São José do Rio Preto, São Paulo, Brazil, 2014-2015

Female sex		
	Resilience Mean [SD (Median)]	Self-esteem Mean [SD (Median)]
Anxiety		
No (n = 28)	130.75[10.93 (132.00)]	20.00[1.72 (20.00)]
Yes (n = 25)	124.48[10.90 (126.00)]	21.68[2.13 (22.00)]
P value*	0.042	0.003
Depression		
No (n = 39)	131.51[9.34 (132.00)]	20.43[1.98 (20.00)]
Yes (n = 14)	117.43[9.74 (118.50)]	21.78[2.12 (22.00)]
P value*	<0.001	0.050

* P value concerning t-test for independent samples at p < 0.05

**Table 5:** Mean±standard deviation (median) of the male participants' resilience and self-esteem scores in regard to anxiety and depression symptoms. São José do Rio Preto, São Paulo, Brazil, 2014-2015

Male sex		
	Resilience Mean [SD (Median)]	Self-esteem Mean [SD (Median)]
Anxiety		
No (n = 53)	132.96[10.09 (135.00)]	19.60[2.19 (20.00)]
Yes (n = 14)	129.79[12.07 (129.00)]	21.14[2.07 (21.00)]
P value*	0.377	0.024
Depression		
No (n = 60)	133.68[10.14 (136.00)]	19.75[2.18 (20.00)]
Yes (n = 7)	120.43[4.83 (121.00)]	21.43[2.37 (21.00)]
P value*	<0.001	0.117

* P value concerning t-test for independent samples at p < 0.05

Women and men with anxiety present higher scores for self-esteem (p = 0.003; p = 0.024) when compared to individuals without the symptoms, as shown in Tables 4 and 5, respectively.

## Discussion

This study's results show that 32.5% and 17.5% of the patients presented anxiety and depression symptoms, respectively. Other studies report similar percentages^14^ ^-^ ^15^, highlighting that the presence of these symptoms may increase the risk of morbidity and mortality, delay hospital discharge, lead to readmission or functional decline, hinder self-care and the adoption of changes necessary to modify one's lifestyle, resulting in non-adherence to treatment^14^ ^,^ ^16^.

Women presented more anxiety and depression symptoms than men, a fact that may be related to women's greater exposure to stressful factors, such as a low socioeconomic level, lack of energy, overload of roles, psychological issues, and low self-esteem. The lower prevalence of these symptoms among men may be explained by a difficulty in reaching a diagnosis, as men tend to deny depression symptoms and compensate with attitudes and behaviors such as anger, aggressiveness, antisocial behavior, excessive consumption of alcohol and hostility^17^. Other studies also identified positive associations between anxiety and depression for the female sex^15^ ^,^ ^17^.

Depression is the main cause of incapacity measured by years of age and the fourth primary contributor to the global burden of diseases; in this study, it is associated with the presence of comorbidities. The prevalence of depression (17.5%) differs from that found in the population in general (approximately 10.0%)^18^. This increase may be attributed to the vulnerability of these patients, who have a chronic condition, to emotional distress, as well as the instrument used, which does not measure the level of depression (mild, moderate or severe), but the presence of depression symptoms.

One study conducted in Greece assessed 190 patients with a diagnosis of heart failure hospitalized in four public hospitals and verified high levels of anxiety (57.3%) and depression (41.6%) measured by HADS^14^.

Various studies indicate the impact negative impact of depression on patients with heart disease^14^ ^-^ ^16^ ^,^ ^19^. One in every five patients presented high levels of depression three months after an episode of myocardial infarction; this mood disorder is associated with increased risk of new cardiovascular event or death^19^. Other studies provide evidence that depression can raise the level of mortality among patients with coronary diseases ^15^ ^,^ ^20^.

The mechanism behind the association of depression with cardiovascular diseases is still unclear, but it is known that it is bi-directional and multi-causal, involving the integration of various central and peripheral processes, causing changes in the immunological system^21^, increased platelet count, inflammation, abnormal heart rate, high catecholamine levels, and endothelial dysfunction^16^. Psychological factors, such as an inability to cope with a chronic disease that presents a high mortality rate and abrupt changes in lifestyle, may also aggravate the condition^16^. 

Individuals identified by HADS as having anxiety and depression symptoms were less resilient than those who did not present symptoms. This association corroborates one German study including 186 patients with congestive heart failure and 372 controls, which verified that resilience is predominantly associated with psychological variables rather than with the severity of disease. The results show that resilience was lower among patients with depression and alexithymia^10^.

Another study that monitored 237,980 men indicates that lower levels of resilience are associated with an increased risk of developing coronary conditions, highlighting that the way in which one faces adversities may be related to lifestyle and environmental factors, such as the practice of exercise, diet, high body mass index, smoking, and social support, and personal characteristics like one's personality^6^.

In regard to sex, this study reveals that men are more resilient than women, a finding that supports the results of a study conducted with individuals with chronic kidney disease^7^. This result may be related to the fact that women more frequently use coping strategies focused on emotions, while men focus on the problem itself^22^, in which an individual opts to solve difficulties and attitudes in order to be able to deal with habitual pressure, decreasing or even eliminating situations that generate stress.

Resilient individuals have greater motivation and ability to solve problems, maintain balance and carry on with their lives with a positive attitude^3^ ^-^ ^4^. The resilience levels of the individuals under study were medium and high, supporting studies conducted in London with patients after surgical correction of tetralogy of Fallot^9^ and in Korea with individuals with congenital heart disease^8^.

Therefore, the nursing team and other health professionals should devise efficient ways to sustain and promote the development of these positive characteristics in the population to improve coping strategies and prepare patients for the unpredictable task of living with a chronic disease.

The scores obtained on the Rosenberg Self-esteem Scale reveal that the feelings people hold about themselves concerning their worth, capacity, importance, and success were moderate (mean score = 20.31 points). 

Women presented higher self-esteem scores when compared to men (p = 0.031). High level of self-esteem was found in a Brazilian study addressing patients with coronary diseases, though no significant difference was found between men and women^23^.

A meta-analysis of 80 studies reports that low self-esteem is a predictor factor for depression, regardless of sex or age^24^. The highest scores for self-esteem in this study were obtained by patients who presented symptoms of anxiety, thereby not corroborating the results reported in the literature^24^. 

Evidence shows that the relationship between depression and self-esteem is more robust that the relationship between anxiety and self-esteem, a fact that may be related to the cognitive vulnerabilities specific to each situation. When depressed, individuals make constant negative assessments of themselves, of the world, of future prospects, and when anxious one anticipates physical or psychological threats^24^. Further studies are needed to verify whether there is an association between anxiety and self-esteem, controlling for variables that may interfere in the process.

Other studies confirm that high self-esteem may be associated with a lower frequency of coronary diseases^20^ and improved quality of life among patients with heart failure^25^. Positive psychological conditions, such as high self-esteem, may protect the heart, preventing systemic inflammation and atherosclerosis, in contrast with negative psychological conditions, such as depression and despair^24^.

Individuals with low levels of self-esteem are more sensitive to the opinions of others and worry over the way other people see or judge them, avoiding exposure and protecting their self-esteem. As a consequence, they may feel lonely, sad, shy, incapable of performing tasks or deriving pleasure from things as they once did, possibly acquiring a negative perception of their own worth, a condition that may lead to the development of symptoms of depression^24^ ^).^

Self-esteem seems to facilitate the prevention of cardiovascular diseases. Therefore, nurses and other healthcare workers should plan an approach focused on the individual. Health assessment focused on the biopsychosocial aspects of a cardiac patient in a hospital environment may contribute to the early identification of anxiety and depression symptoms, low self-esteem and impaired resilience, which can aid the promotion of individual or group strategies to treat these conditions.

This study was limited by time and its cross-sectional design, such that long-term follow-up that could enable assessing resilience and self-esteem in each phase of the cardiovascular disease was not possible. Additionally, only those patients admitted to the hospital chosen for this study were included, hence, these results cannot be generalized to other clinical settings.

Nevertheless, this study's results enabled understanding some relevant emotional characteristics of patients with cardiovascular diseases, so that nurses and other healthcare professionals can expand their work possibilities, with interventions focused on the psychosocial conditions of patients, helping them to deal with their disease in a better way by adopting a more positive lifestyle. Additionally, the results show the need for future clinical studies to test programs designed to strengthen cognitive and emotional skills, producing new scientific evidence to implement in practice.

## Conclusion

In conclusion, this study's results present some psychosocial characteristics of patients with cardiovascular diseases. Women more frequently present anxiety and depression symptoms and higher self-esteem, while men are more resilient.

Patients of both sexes with depression symptoms and women with anxiety were associated with lower scores for resilience. Self-esteem levels were higher among men and women with anxiety.

This study has important implications for clinical practice and for nursing research, as it points out ways to improve approaches for patients in a hospital setting and directs future studies addressing interventions intended to improve coping with adversities and promoting a positive perception of oneself. 
